# Microbial Diversity for Biotechnology

**DOI:** 10.1155/2014/845972

**Published:** 2014-01-23

**Authors:** George Tsiamis, Dimitrios Karpouzas, Ameur Cherif, Konstantinos Mavrommatis

**Affiliations:** ^1^Department of Environmental and Natural Resources Management, University of Patras, 2 Seferi Street, 30100 Agrinio, Greece; ^2^Department of Biochemistry and Biotechnology, University of Thessaly, Ploutonos 26 and Aiolou Street, 41221 Larisa, Greece; ^3^Higher Institute for Biotechnology, University of Manouba, Biotechpole of Sidi Thabet, Sidi Thabet, 2020 Ariana, Tunisia; ^4^DOE Joint Genome Institute, 2800 Mitchell Drive, Walnut Creek, CA 94598, USA

The aim of this special issue is to emphasize on the ecology of microorganisms, the most diverse and abundant group of organisms on Earth, and to infer on biotechnological applications. Even though we live in a microbially dominated planet only the past decade the study of the microbial diversity has entered a period of considerable importance to science in general, industry, protection of the environment, and public policy making. Environmental microbes are immensely diverse and have numerous metabolic activities and products that could have industrial applications. This treasured reservoir is largely unexploited since more than 99% of environmental microbes cannot be cultured under current laboratory conditions, leaving their potential largely unused. Understanding the unculturable fraction of Earth's microbiome is essential to understand the evolution, sustainability of life on Earth, and the development of various industrial products that have potential applications across all major industries.

This special issue contains seven reviews and eighteen research articles that provide a better understanding (a) of the hidden microbial diversity and (b) the use and development of new technologies that can lead to potential biotechnological applications.

K. A. Kormas and D. S. Lymperopoulou presented a review article on the meta-analysis of the 16S rRNA and *mlrA* (microcystinase) gene diversity of isolates that are known to degrade cyanobacterial toxins. Members of *Proteobacteria*, *Arthrobacter*, *Bacillus*, and *Lactobacillus* have been found as potential degraders of the toxins produced by *Cyanobacteria*.

S. Nikolaki and G. Tsiamis presented a comprehensive overview of the advanced tools that became available in the last few years to the microbial ecologist and describe recent examples and future applications of such tools including phylogenetic and functional microarrays, next generation sequencing, and single cell genomics. These tools will enable exploitation of the previously unknown microbial diversity in biotechnology.

O. Selama et al. attempt to correlate the presence of common bacterial phyla to geographical regions (countries/continents) using the NCBI nucleotide database to get microbial data accessions with a country qualifier set and compare with the global biodiversity information facility (GBIF) database data set. This work emphasizes the importance of metadata tracking and submission in a standardized and enforced fashion and highlights the limitations of resources such as NCBI and GBIF as well as the biased sampling throughout the years, which limits an unbiased understanding of bacterial biodiversity and geographical abundance.

S. Ntougias et al. provided a detailed review of the microbial communities identified over the past 20 years in olive mill wastes (OMW) using both culture-dependent and culture-independent approaches. They also discuss OMW-induced toxicity and the effects that the OMW have on soil microbiota. Finally, it is presented in an elegant way the biotechnological importance of the OMW microbiota with respect to (a) the biodegradation of OMWs, (b) the bioconversion aspects of the OMW microbiota, and (c) the plant-disease-suppressive properties of OMW.

D. Trabelsi and R. Mhamdi report on the effect that the release of microbial agents could have on indigenous soil microbial communities and provide an extensive review on the most significant studies addressing the impact of inoculation on soil microbial communities. The authors conclude that the assessment of the observed impacts depends largely on the techniques used to address the dynamics of the soil microbial communities.

Three papers by L. Berg et al., A. K. Chaudhary et al., and P. Jeandet et al. add new insights into the concept of microbial cell factories and their exploitation as platforms for the biosynthesis of numerous molecules. The first paper describes the application of a combinatorial mutagenetic approach, which resulted in the development of variants of the *P*_*AOX1*_ promoter of the yeast *Pichia pastoris* with increased expression levels and abolished glucose repression. The second paper provides an overview of the application of omic approaches into the engineering of *Streptomyces* aiming to augment their industrial exploitation in the production of secondary metabolites. Pitfalls that have hampered the use of *Streptomyces* as cell factories for the production of antibiotics are described and current examples and future trends on the use of omics in the genetic improvement of this Actinobacteria group are presented. The third paper gives an overview of the recent progresses that have been made for the last 10 years in metabolic engineering of microorganisms for the biosynthesis of natural products of pharmaceutical significance.

Two papers report on the complexity of the microbial interactions. L. Vanysacker et al. following a multiphasic approach demonstrated the complexity of the microbial interactions controlling biofilm formation on microfiltration membranes used in wastewater treatment facilities. Their study elegantly demonstrated that it is mostly the composition of the microbial community and not the type of membrane that drives the biofouling process. C. Díaz et al. studied the behaviour of yeasts during spontaneous wine fermentation using culture-dependent and culture-independent approaches. They report that the yeast diversity in musts from red grape was greater than the one recorded from white grape varieties. Interestingly, yeast quantification indicated that non-*Saccharomyces* yeasts were present during the entire fermentation process with *R. mucilaginosa* and *P. anomala* being the most prominent species.

Three papers by L. Bifulco et al., M. Stathopoulou et al., and I. Fhoula et al. report on the exploitation of bacterial and gene diversity for the development of potential biotechnological applications. The first paper presents a first evidence for the development and preliminary validation of an electrochemical genosensor, which could be used as a rapid diagnostic tool for the detection of pathogenic *Listeria monocytogenes* over nonpathogenic *Listeria* strains. This is based on the detection of the *inlA* gene encoding an 80 kDa surface protein which allows *Listeria* to enter the cells. The second paper by P. Stathopoulou et al. examined the lipolytic profile of 101 bacterial strains isolated from the volcanic area of Santorini, Aegean Sea, Greece. Nine bacterial strains exhibiting extracellular lipase activities were characterized as *Aneurinibacillus *sp. with an optimum activity at 70–80°C (pH 8-9). The isolated lipases revealed exceptional thermostability with high optimum activity temperatures, and they represent very promising candidate enzymes for a variety of high temperature industrial lipolytic applications. The third paper by I. Fhoula et al. examined the biotechnological properties of lactic acid bacteria isolated from the rhizosphere of olive trees and desert truffles. Rhizospheric strains that belong to *Enterococcus* and *Weissella* exhibited strong antibacterial activity against plant pathogenic and food-borne bacteria.

The papers by I. Maeda et al., C. Hamdi et al., and A. Rizzi et al. examined the diversity of gut microbes. More specifically I. Maeda et al. present a study of gut microorganisms from three jungle crows using 16S rRNA-targeted sequencing which reveals the presence of potentially pathogenic bacterial genera. These findings indicate the importance of crows in public health and their role as natural reservoirs of pathogenic microorganisms. C. Hamdi et al. examined the diversity of *Paenibacillus larvae*, the causative agent of American foulbrood (AFB). BOX-PCR fingerprints indicated relatively high intraspecific diversity among the *P. larvae* isolates with six genotypes being identified. A. Rizzi et al. characterized the gut microbial communities that are associated with the wood-boring beetle *Anoplophora chinensis* using culture-dependent and culture-independent approaches. Mainly *Proteobacteria*, *Actibobacteria*, and *Firmicutes* dominated the bacterial diversity with the most dominant taxa being the *Enterobacteriaceae* family of Gammaproteobacteria.

Three papers by D. El Hidri et al., C Moulas et al., and L. Ma et al. studied the diversity of microbes in different ecosystems. D. El Hidri et al. deployed a culture-dependent approach to study the diversity and ecology of haloalkaliphilic bacteria in arid saline ecosystems from Southern Tunisia. 122 haloalkaliphilic strains were isolated with thirteen genera and twenty distinct species being identified. In the second paper C. Moulas et al. examined the impact of two systemic pesticides on the epiphytic fungal and bacterial communities via DGGE and cloning. The fungal community was dominated by putative plant pathogenic ascomycetes, with yeast isolates of *Cryptococcus* sp. being stimulated by the application of imidacloprid, suggesting a potential role in its degradation. In the third paper the composition and dynamics of planktonic viruses and their relationship with environmental parameters in natural freshwater were examined. Correlation analysis indicated that the main factor influencing viral abundance is bacteria.

Two papers by M. S. Silva et al. and G. S. Kanini et al., addressed the Actinobacterial diversity and their potential biotechnological applications. In the first paper the diversity of *Actinobacteria* spp from three Brazilian Savannah soils was examined. All sites exhibited a high *Streptomyces* diversity and interestingly enough there were no significant differences in the concentrations of phosphorus, magnesium, and organic matter in the soils. Samples from the rainy season exhibited higher Actinobacterial diversity than the samples from the dry season. In the second paper the bacterial strain ACTA155, member of *Streptomyces* genus isolated from diverse Greek habitats, exhibited strong antifungal activity against the phytopathogenic fungus *Fusarium oxysporum*. Interestingly, the metabolites involved in the antagonistic action of the isolate showed to be more than one and produced independently of the presence of the pathogen which further highlighted the biotechnological potential of this isolate.

Two papers by A. Krimitzas et al. and I. B. Fekih et al. examined fungal diversity. In the first paper the diversity of *Aspergillus* species using morphological and molecular criteria is presented. In very diverse genera like *Aspergillus* the use of single gene-based analyses does not solve all ambiguities and does not always represent the evolutionary history of the species. Utilization of nuclear and mitochondrial genes enabled the authors to fully characterize more than thirty-five *Aspergillus* strains in sixteen sections. In the second paper the diversity of Entomophthoralean fungi was investigated using morphological and molecular (ITS) markers in the aphid species of *Sitobion avenae* and *Entomophthora planchoniana,* with the fungal species of *Pandora neoaphidis* and *Entomophtra planchoniana *being the most dominant.

The role of plant growth promoting (PGP) bacteria was examined in two papers. R. Marasco et al. examined the environmental factors that influence the PGP potential of the root-associated bacteria with the grapevine root system from Egypt, Tunisia, and Northern Italy. PCR-DGGE analysis indicated that the structure of endospheric and rhizospheric bacterial communities was highly diverse and was associated with a cultivar/latitudinal/climatic effect. The microbial profile of the Tunisian grapevines was more similar with the Tunisian than those cultivated in Northern Italy. In the second paper F. Mapelli et al. characterized halophilic/halotolerant bacteria from the rhizosphere of *Salicornia* plants and bulk soil collected from hypersaline systems in Tunisia. The biotechnological potential of these isolated bacterial strains was further highlighted when at least twenty *Halomonas* strains displayed PGP features *in vitro* and were able to colonize *Salicornia* roots under laboratory conditions.

Collectively, these papers give a comprehensive view on the immense potential that the study of microbial diversity can provide towards the development of biotechnological applications. Current and ongoing advances in the field of omic approaches will certainly propel our capacity to understand and further exploit microbial diversity. In this regard, these articles provide a prospective into the future of this field.

